# Acute Pancreatitis Secondary to Cocaine Intoxication: A Case Series

**DOI:** 10.7759/cureus.58420

**Published:** 2024-04-16

**Authors:** Oxana Ushakova, Keyvan Ravakhah

**Affiliations:** 1 Internal Medicine, MetroHealth Medical Center, Cleveland, USA; 2 Internal Medicine, Mount Carmel Medical Center, Columbus, USA; 3 Internal Medicine, St. Vincent Charity Medical Center, Cleveland, USA

**Keywords:** clinica gastroenterology, gastroenterology, toxicology and poisoning, cocaine toxicity, pancreatitis

## Abstract

Gallstones, alcohol use, hypertriglyceridemia, and hypercalcemia have been considered the most common causes of acute pancreatitis; however, about 20% of the cases remain idiopathic since no definite cause can be established. It has been noticed that there is a small number of patients who have presented to the hospital with a diagnosis of acute pancreatitis who have concurrently been using cocaine yet have no recent alcohol use and no gallstones. The purpose of this series of case reports is to review the evidence behind the association between cocaine and pancreatitis. In most of the cases, the etiology of acute pancreatitis is usually straightforward. However, when faced with a patient who has acute pancreatitis but lacks the common causes such as alcoholism, gallstones, normal triglyceride levels, and no evidence of malignancy, it seems reasonable to consider drugs as a potential cause for pancreatitis.

## Introduction

As of 2020, global estimates indicate that approximately 5.2 million individuals annually (1.9% of the population aged 12 and above) engage in cocaine use [1]. Cocaine use has been consistently linked with severe medical complications [2]. Among the most prevalent are cardiovascular effects, encompassing disruptions in cardiac rhythm and incidents of myocardial infarction [3,4]; neurological manifestations, including but not limited to headaches, seizures, cerebral vascular accidents, and states of coma [5]; and gastrointestinal disturbances, such as abdominal pain and emetic symptoms [6-12]. Notably, instances of sudden death have been documented, with fatalities often attributed to cardiac arrhythmias or convulsive episodes [3].

Recent studies have provided further insights into the epidemiology of cocaine use and its associated risks. A longitudinal cohort study published in 2023 reported a global increase in the prevalence of cocaine use among young adults aged 15-64, highlighting shifting trends in drug consumption patterns [13]. Moreover, a review analysis published in 2022 identified a significant association between cocaine use and the development of cardiovascular complications, emphasizing the need for targeted interventions and risk mitigation strategies [14].

A thorough examination of English literature revealed a limited number of case reports documenting instances of cocaine-induced pancreatitis [15]. The primary objective is to conduct a comprehensive review elucidating the association between cocaine consumption and pancreatitis.

## Materials and methods

By utilizing an International Classification of Diseases (ICD) code for acute pancreatitis, we identified 572 patient charts at St. Vincent Charity Medical Center within a five-year span between 2012 and 2017 bearing the diagnosis of acute pancreatitis. Subsequently, we reviewed these records for an ICD code indicative of cocaine intoxication. Patients with pancreatitis diagnosed due to gallstones, alcoholism, hypercalcemia, or hypertriglyceridemia were excluded from our analysis. Our scrutiny revealed six individuals with urine toxicology positive for cocaine, devoid of any other discernible causes for pancreatitis. Diagnosis of pancreatitis was established following current guideline criteria, which encompassed elevated levels of amylase and lipase (at least three times the normal levels), corroborated by CT scan findings and classic clinical manifestations, such as abdominal pain, fever, anorexia, nausea, and vomiting. Only patients with a definitive diagnosis of acute pancreatitis were included in our study.

Employing an ICD code for cocaine intoxication or use disorder, we identified 186 patients within the same timeframe. Notably, all six individuals with acute pancreatitis were encompassed within the cocaine intoxication group. Our control group comprised admissions devoid of both pancreatitis and cocaine abuse.

Inclusion criteria for cocaine-associated acute pancreatitis: all patients admitted with acute pancreatitis who underwent a toxicology screen during the same admission, testing positive for cocaine use, irrespective of age, race, or gender, and with no other known etiology for acute pancreatitis.

Exclusion criteria for cocaine-associated acute pancreatitis: patients with a history of alcohol consumption, regardless of quantity, active cholelithiasis, triglyceride levels exceeding 500 mg/dL, corrected calcium levels surpassing 11 mg/dL; those currently taking medications such as antiretrovirals and thiazide diuretics or medications associated with multiple documented causes of acute pancreatitis; those with any active malignancy; and those who underwent recent endoscopic retrograde cholangiopancreatography (ERCP) procedures if documented.

Data analysis method

The table presenting counts and percentages (%) of pancreatitis cases was juxtaposed between groups (positive versus negative for cocaine use) utilizing the chi-square test, with Yates’ correction for continuity, to evaluate the relative occurrence frequency of pancreatitis. Statistical significance was set at p < 0.05. The predictive power of the group classifier (positive versus negative for cocaine use) on pancreatitis occurrence was demonstrated by estimating the odds ratio (OR) and its 95% confidence interval (95% CI). Additionally, the distribution of ages and genders among patients was examined. Gender data were summarized in counts and percentages (%), whereas quantitative age data were described using sample mean, sample standard deviation (SD), and range (minimum and maximum), alongside median, 25th, and 75th percentile values. The statistical analysis was executed utilizing Statistical Product and Service Solutions (SPSS, version 22; IBM Corp., Armonk, NY) software.

## Results

Our study uncovered some significant differences in the occurrence of pancreatitis between patients who use cocaine and those who do not. Specifically, we found that 3.22% of patients who used cocaine experienced pancreatitis, whereas only 1.16% of non-cocaine users had pancreatitis, as illustrated in Table 1. This discrepancy was not merely due to chance, as our statistical analysis revealed a p-value of 0.0228, indicating statistical significance. We utilized the chi-square test of contingency table data with Yates’ correction for continuity to determine this significance. Furthermore, the OR for pancreatitis among cocaine users was calculated to be 2.84 (95% CI: 1.25-6.44). The CI did not include 1.00, emphasizing the substantial difference in pancreatitis frequency between the two groups. Notably, the upper limit of the confidence interval, reaching 6.44, indicates the potential for further investigation in future studies to explore this relationship in more depth.

**Table 1:** Cross-tabulation of cocaine use and pancreatitis (counts: percentage) Chi-square with Yates' correction Chi-squared equals 5.187 with one degree of freedom; the two-tailed P value equals 0.0228. Odds ratio: 2.84, 95% CI: 1.25-6.44

Groups	Cocaine users, number (%)	Non-users, number (%)	Total, number (%)
Pancreatitis	6 (3.22%)	566 (1.16%)	572 (1.17%)
Controls	180 (96.78%)	48,263 (98.84%)	48,443 (98.83%)
Total	186 (100%)	48,829 (100%)	49,015 (100%)

In addition to examining the occurrence of pancreatitis, we analyzed demographic characteristics, such as gender and age, among the study participants, as detailed in Tables 2-3. Despite the challenge posed by the limited number of acute pancreatitis cases, we observed some interesting trends. For instance, we noted that the percentage of females with pancreatitis slightly exceeded those without, suggesting a potential gender-related aspect to the condition. Furthermore, individuals diagnosed with pancreatitis tended to be older than those without the condition. Specifically, the data revealed a narrow age range among individuals with acute pancreatitis, with a predominant age group in their 50s.

**Table 2:** Gender distribution of cocaine users, pancreatitis versus controls

Gender	Pancreatitis, number (%)	No pancreatitis, number (%)	All cocaine users, number (%)
Male	3 (50%)	134 (74.44%)	137 (73.66%)
Female	3 (50%)	46 (25.56%)	49 (26.34%)
Total	6 (100%)	180 (100%)	186 (100%)

**Table 3:** Age (years) distribution of cocaine users, pancreatitis versus controls

Descriptive statistics tools	Pancreatitis, number	No pancreatitis, number	All cocaine users, number
Mean	54.80	50.23	50.36
Standard deviation (SD)	2.59	9.46	9.37
Count	6	180	186
Median	54.00	51.00	51.00
First quartile	54.00	44.75	45.00
Third quartile	55.00	57.00	57.00
Maximum	59	71	71
Minimum	52	24	24

## Discussion

The results of our study suggest a significant association between acute cocaine use and the occurrence of acute pancreatitis. Our analysis revealed that the relative frequency of pancreatitis was notably higher among patients who tested positive for cocaine compared to those without a history of cocaine use. The statistical analysis, including the chi-square test and calculation of OR, further confirmed this association, with a p-value of 0.0088 and an OR of 2.84, indicating that individuals testing positive for cocaine are nearly three times more likely to develop pancreatitis compared to non-cocaine users. These findings underscore the importance of recognizing cocaine as a potential risk factor for pancreatitis. In the other articles, there are at least seven reported cases of acute pancreatitis happened in young patients within 48 hours of cocaine exposure without active alcohol use or other known risk factors [6], in whom no further recurrent pancreatitis episodes were reported upon cocaine use cessation, which also supports our observations.

However, our study faced certain limitations that should be acknowledged. One significant limitation was the reliance on electronic medical records, which only capture cocaine use through ICD coding, leading to potential underestimation of actual cocaine use within the hospital population. Additionally, the inability to accurately ascertain the true number of cocaine users due to the lack of data from positive toxicology screens posed a challenge. This limitation underscores the need for alternative methods, such as patient surveys, to supplement existing data sources and provide a more comprehensive understanding of cocaine prevalence.

To address these limitations and further elucidate the association between cocaine use and pancreatitis, future research endeavors should consider employing a large multicenter study design. Such a study could leverage electronic medical record systems offering greater flexibility in data mining parameters and incorporate toxicology reports to enhance accuracy. Additionally, conducting patient surveys at specific intervals to gather information on recent cocaine use could provide valuable insights. By overcoming these methodological challenges, future studies may uncover additional evidence supporting the observed association and elucidate potential mechanisms underlying cocaine-induced pancreatitis. While our study provides preliminary evidence of a link between acute cocaine use and pancreatitis, further research is warranted to confirm and expand upon these findings. Addressing the limitations of our study design through larger, more comprehensive investigations will be essential in advancing our understanding of the relationship between cocaine use and pancreatitis and informing clinical practice and public health interventions.

## Conclusions

Our observations suggest a potential association between cocaine use and acute pancreatitis, although this phenomenon remains rare and is not extensively documented in the literature. The proposed mechanism of action, involving vasoconstriction and platelet activation leading to a vaso-occlusive disorder, provides a plausible explanation for this link. However, further research is needed to fully understand the intricate cause-and-effect relationship between cocaine use and pancreatitis. Such studies are crucial for advancing our knowledge in this area and developing effective prevention and treatment strategies to mitigate the risks associated with cocaine-induced pancreatitis.
